# Supplementary material for: A set of multi-entry identification keys to African frugivorous flies (Diptera, Tephritidae)
Source: Zookeys. 2014 Jul 24;(428):97–108. doi: 10.3897/zookeys.428.7366 (PMC4143993; doi:10.3897/zookeys.428.7366)
Supplement: Supplementary material 6 — Key to Ceratitis [file zookeys-428-097-s006.zip › SF6_ZooKeys_key to Ceratitis/key/SF6_ZooKeys_key to Ceratitis/Media/Html/Ceratitis caetrata.htm]

Ceratitis (Ceratitis) caetrata Munro


***Ceratitis*** **(*Ceratitis*) *caetrata*
Munro**

*Ceratitis* (*Ceratitis*) *caetrata*
Munro, 1949: 499.

Body
length: 4.02 (3.90-4.20) mm; wing length: 4.31 (4.00-4.65) mm.

Male

Head.
Antenna yellow. Third antennal segment twice as long as second segment. Arista
with short hairs. Frons yellow, inflated above antennal implant; almost bare,
at most with few short scattered hairs which are largely the same colour as
frons. Ocellar and orbital bristles yellow; lower orbital modified, apical end
dark and fan shaped, at least twice as long as arista; upper orbital much
reduced, weakly developed. Frontal bristles absent. Face yellow-white. Genal
bristle pale, genal setulae dark reddish, weakly developed. Postocellar and
outer vertical bristles pale.

Thorax.
Postpronotum pale yellow, with distinct black spot. Mesonotum: ground colour
orange; microtrichiae pattern silvery with orange shine, spots brown or
black-brown; sutural spots whitish, small; prescutellar whitish markings
separate. Scapular setae pale. Scutellum yellow-white, basally with two dark
separate spots, apically with three merged spots, incisions variable, at least
till halfway and sometimes almost reaching apical bristles. Anepisternum
completely pale. 

Legs.
Yellow; setation typical for subgenus, mainly pale especially on femora. Fore
femur posteriorly with bush of longer orange-coloured hairs along entire
length, posterodorsal hairs longer; ventral spines yellow-orange; anteroventral
row of hairs short and yellow. Hind femur with longer hairs dorsally and
ventrally on apical fourth. 

Wing.
Marginal band with clear and complete interruption; cubital band free; medial
band absent; crossvein r-m at middle of discal cell. Vein R1 beyond crossvein
r-m. Crossvein dm-cu posteriorly more outwards than anteriorly.

Abdomen.
Yellow. Setation and banding typical for subgenus.

Female

As
in male except for the following characters: Third antennal segment two to
three times as long as second segment; yellow-orange.  Frons yellow-orange,
only slightly inflated; two frontal bristles present; short hairs more
developed and distinctly darker than frons. Ocellars dark (frontal and orbitals
presumably also but missing in specimens studied). Orbitals not modified
(according original description). Genal setulae strongly developed. Mesonotal
pattern sometimes much darker, more like in *C. capitata*. Crossvein dm-cu
posteriorly more inwards. Legs without feathering. Oviscape shorter than
abdominal terga.

(Description
after De Meyer, 2000)
